# Long term quality of life after laparoscopic antireflux surgery for the elderly

**DOI:** 10.1186/1471-2482-13-S2-S10

**Published:** 2013-10-08

**Authors:** Salvatore Tolone, Giovanni Docimo, Gianmattia Del Genio, Luigi Brusciano, Ignazio Verde, Simona Gili, Chiara Vitiello, Antonio D'Alessandro, Giuseppina Casalino, Francesco Saverio Lucido, Nicola Leone, Raffaele Pirozzi, Roberto Ruggiero, Ludovico Docimo

**Affiliations:** 1 Division of General and Bariatric Surgery, Department of Surgery, Second University of Naples, Via S. Pansini 5, 80131, Naples, Italy

## Abstract

**Background:**

Studies have previously shown laparoscopic antireflux surgery is a safe and effective treatment for GERD even in elderly patients. The aim of the current study was to evaluate patients receiving laparoscopic antireflux surgery before and after 65 years of age and to assess their surgical outcomes and improvements in long term quality of life.

**Methods:**

Patients were given a standardized symptoms questionnaire and the Short-Form 36 Health Survey for quality-of-life evaluation before and after laparoscopic total fundoplication.

**Results:**

Forty-nine patients older than 65 years of age were defined as the elderly group (EG) whereas the remaining 262 younger than 65 years of age were defined as the young group (YG).

There were 114 (36.6%) patients who filled out the SF36 questionnaire (98 in the younger group, rate: 37.4%; 16 in the elderly group, rate: 32.6%) pre- and post-operatively. There was no significant difference between the two age groups regarding preoperative PCS ( 45.6 ± 7.8 in YG vs. 44.2 ± 8.2 in EG; P = 0.51) and MCS ( 48.1 ± 10.7 in YG vs. 46.9 ± 9.2 in EG; P = 0.67). There was no significant difference between the two age groups regarding postoperative PCS (49.8 ± 11.9 in YG and 48.2 ± 9.5 in EG ; P = 0.61 and MCS (48.4 ± 10.7 in YG vs. 50.1 ± 6.9 in EG; P = 0.54).

**Conclusions:**

In conclusion, laparoscopic total fundoplication is a safe and effective surgical treatment for gastroesophageal reflux disease generally warranting low morbidity and mortality rates and a significant improvement of symptoms comparable. An improved long-term quality of life is warranted even in the elderly.

## Background

Gastroesophageal reflux disease is a frequent chronic disease related to retrograde flow of gastroduodenal contents into the esophagus [1]. As long-term therapeutic strategies for chronic gastro-esophageal reflux disease (GERD), surgery and Proton-pump inhibitors are effective and well tolerated, with antireflux surgery being superior in controlling overall disease manifestations [2]. Only 20 years have passed since the introduction of the first laparoscopic cholecystectomy and its general acceptance as the standard treatment for patients with benign and malignant disease [3-7]. Since then, a great number of different surgical procedures has been carried out via laparoscopy [8-11] Laparoscopic surgery for GERD carries significant advantages in terms of lower overall morbidity, shorter postoperative hospital stay, and faster return to normal physical activity [12]. The measurement of quality of life ( QoL) is of paramount importance when attempting to compare outcomes after treatments, because neither questioning the patient about symptoms nor the assessment of objective instrumental testing seem to adequately provide an adequate assessment of patients subjective wellbeing [13].

Studies have previously shown laparoscopic antireflux surgery is a safe and effective treatment for GERD even in elderly patients, warranting low morbidity and mortality rates and a significant improvement of symptoms comparable to younger patients [14,15].

The aim of the current study was to evaluate patients receiving laparoscopic antireflux surgery before and after 65 years of age and to assess their surgical outcomes and improvements in long term quality of life.

## Methods

A prospective electronic database of all patients admitted for GERD in our university medical center ( Division of General Surgery, Second University of Naples, Naples, Italy), was reviewed. Demographic data were obtained at the time of first visit. Patients older than 65 years of age were defined as the elderly group (EG) whereas the remaining as the young group (YG).

The study protocol was approved previously by the Ethical Committee of the Second University of Naples. All patients and controls were Caucasians from Italy.

Patients having typical or atypical symptoms for at least 6 months and requiring daily medical therapy for symptom control were offered the alternative of continuing with medical therapy or undergoing antireflux surgery. Patients who had undergone previous antireflux surgery or who required a concurrent abdominal procedure at the same time as fundoplication (eg, cholecystectomy), as well as patients with Barrett's esophagus, were excluded from this study.

All patients underwent a scheduled work-up that included upper- gastrointestinal endoscopy, barium meal radiograph examination, stationary esophageal manometry, 24-hour ambulatory pH monitoring and since available also 24 hours Combined Multichannel Intraluminal Impedance and pH-monitoring (MII-pH) as well as Combined Multichannel Intraluminal Impedance and Esophageal Manometry (MII-EM) or High Resolution impedance Manometry (HRiM) [16].

After a brief interview and examination to assess for presence and severity of gastrointestinal symptoms and to make anthropometric measurements, all subjects completed a brief symptom assessment questionnaire. The questionnaire incorporated a visual analog scale (modified DeMeester Score) for heartburn, regurgitation and atypical symptoms [14].

Quality of life was evaluated using the Italian language Short-Form 36 (SF-36)[17,18].

This is a generic measure of perceived health status, widely used in medical and health service research, that incorporates behavioral functioning, subjective well-being, and perception of health by assessing 8 health concepts: physical function, role-physical, bodily pain, general health, vitality, social function (SF), role emotional, and mental health. The Italian translation of this questionnaire has been validated previously using objective psychometric criteria.14 Scores range from 0 to 100, with higher scores indicating better functioning and well-being. Ware et al15 developed Physical Component Summary (PCS) and Mental Component Summary (MCS) scores based on the SF-36 scales. The PCS is mostly the result of the following health concept: physical function, role-physical, and bodily pain. The MCS is mostly the result of the following health concept: SF, role-emotional, and mental health. Three of the scales (vitality, general health, and SF) have noteworthy correlations with both components. The 2 sets of summary scores have been proven to provide reliable evidence of medical well-being in the US general population; a 3-point difference in the scores is regarded as clinically important. All patients underwent a laparoscopic total fundoplication. An antibiotic prophylaxis was administered preoperatively[19].

The rate of complications and death events was recorded[20].

Briefly, patient is placed in a lithotomy position with legs abducted. Five trocars are placed as previously described. The gastric fundus is mobilized well, dissecting the fold of the peritoneal reflection and completely resecting the gastrophrenic ligament without dividing the short gastric vessels. After exposing the esophageal anterior wall and identifying the anterior vagal nerve, surgical dissection by ultrasonic dissection is accomplished[21-23].

The diaphragmatic crus is divided and the lower mediastinal and abdominal portion of the esophagus is mobilized. Taking care to avoid injury to the posterior vagal nerve, a wide retroesophageal space is created and the esophagus is retracted gently retracted to approximate the crura with 1 nonabsorbable stitch. Then a 360° antireflux wrap is constructed by suturing 2 gastric hemivalves with 2 nonabsorbable stitches placed 20 mm apart. The suture does not include the esophageal wall, diaphragmatic pillars, or lesser gastric curve. Then, the endoscope is reinserted to check the morphologic aspect of the fundoplication. The use of hemostatic agents was employed when necessary[24].

When applicable, patients underwent protocols consistent with previous studies[25,26].

All patients were contacted by telephone or mail at least 1 year after laparoscopic total fundoplication and invited to came and answer the same questionnaires administered preoperatively. Satisfaction of the procedure and the will of undergoing the same operation after knowing its effects were defined as excellent outcome [27]. Statistical analysis was performed on the patients who completed the postoperative evaluation using SPSS for Windows (version 17.0; SPSS, Inc., Chicago, IL). Results were expressed as mean ± SD unless otherwise indicated. Continuous variables were compared by the unpaired and paired Student *t*test and dichotomic variables were compared by the chi-squared test and the McNemar test. The Wilcoxon signed-rank sum test was used to compare the preoperative and postoperative symptom severity score. The data were analyzed by Mann-Whitney *U*tests to test for outcome differences between young and older patients. P value < 0.05 was considered statistically significant.

## Results

From September 2001 to December 2011, 311 consecutive patients, 127 male and 184 female, mean age 45.8 years (range 12-80) with GERD underwent laparoscopic Nissen- Rossetti fundoplication.

Forty-nine patients older than 65 years of age were defined as the elderly group (EG) whereas the remaining 262 younger than 65 years of age were defined as the young group (YG).

Demographics data and ASA score of the two groups are listed in Table 1.

**Table 1:** Demographics

	EG (n = 49)	YG (n = 262)	p-value
Age	73.2 ± 1.8	49.2 ± 3.5	*0.0001*
Gender(Male)	13	73	*0.88*
ASA score	2.3 ± 0.5	2.4 ± 0.6	*0.273*
BMI	24.9 ± 3.7	24.7 ± 3.3	0.69

All the interventions were completed *via*laparoscopic approach. Mean operative time was 53 ± 12 min in YG and 64.6 ± 17 min in EG. No mortality was observed in both groups. A major complication occurred in 4/262 patients (1.5%), all among the YG. Blood loss was lower in YG group(*P *= 0.0001.) Mean postoperative hospital stay was longer in the EG group (*P *= 0.0001) (Table 2). Normal activity resumed in 8.4 ± 3.7 days in YG and 12.1 ± 7.5 days in EG (*P =*0.0001)

**Table 2:** Surgical outcomes

	EG (n = 49)	YG (n = 262)	p-value
OR time(hr)*	64.6 ± 17	53.9 ± 12	*0.0001*
EBL	53 ± 17	34 ± 13	0.0001
Complications	0	4	*0.38*
LOS(d)‡	3.6 ± 1.4	2.8 ± 1.3	*0.0001*

*OR, operative time; †EBL, estimated blood loss; ‡LOS, length of hospital stay.

At the follow-up evaluation (mean, 48.1 ± 12.4 mo; range, 12-84 mo) antireflux surgery significantly diminished the symptom severity score of heartburn, epigastric pain, regurgitation, and respiratory symptoms in both groups (*P*< 0.05). Persisting postoperative dysphagia (DeMeester score 2-3) leading to > 15% of weight loss was observed in 10 (4%) of 262 patients in YG and in 2 (4%) of 49 patients. An excellent outcome was observed in 92/98 (94%) younger patients and in 15/16 (93.7%) elderly patients (*P = 0.98*)..

## Quality of life

There were 114 (36.6%) patients who filled out the SF36 questionnaire (98 in the younger group, rate: 37.4%; 16 in the elderly group, rate: 32.6%) pre- and post-operatively.

Table 3 shows the 8 SF-36 scale scores for patients in the preoperative and postoperative evaluation either in younger group (YG) and elderly group (EG) patients. A significant improvement in 4 of 8 scale score between the preoperative and postoperative evaluation both in the younger and elderly group has been observed. Comparisons of the preoperative and postoperative SF 36 summary scores in the two age groups were made.

**Table 3:** Comparison of the preoperative and postoperative SF-36 scale scores

SF-36 scales	EG (n = 16) preop	YG(n = 98)preop	EG (n = 16) postop	**YG(n = 98)postop**.
Physical function	65.5 (18.4)†	73.3 (19.2)‡	78.3 (16.0)	85.1 (22.6)
Role limitations-physical	56.4 (21.6)†	59.4 (22.9)‡	79.4 (18.7)	82.9 (26.9)
Bodily pain	52.5 (19.3)†	55.5 (19.1)‡	65.6 (14.1)	67.9 (27.8)
General health perception	60.2 (23.4)	62.1 (21.2)	62.8 (18.3)	65.5 (18.2)
Vitality	46.2 (15.3)†	48.7 (15.9)‡	58.6 (17.5)	61.5 (20.9)
Social function	69.4 (18.1)	71.2 (19.9)	71.0 (12.5)	74.6 (21.9)
Role limitations-emotional	66.8 (19.9)	69.4 (20.6)	75.1 (16.0)	73.1 (34.5)
Mental health	60.1 (16.1)	67.1 (16.7)	62.4 (15.8)	65.3 (19.4)

All data reported as mean (SD).

preop= preoperative; postop=postoperativeHC _ healthy controls

*All preoperative an postoperative values were similar between EG and YG (*P = NS)*.

†*p <*.05, preoperative vs postoperative in EG group (mean follow-up period, 48.1 ± 12.4 mo).

‡*p <*.05, preoperative vs postoperative in EG group (mean follow-up period, 48.1 ± 12.4 mo).

There was no significant difference between the two age groups regarding PCS ( 45.6 ± 7.8 in YG vs. 44.2 ± 8.2 in EG; *P = 0.51*) and MCS ( 48.1 ± 10.7 in YG vs. 46.9 ± 9.2 in EG; P = 0.67).

Similar results were also revealed in a comparison of the postoperative SF 36 summary scores. The postoperative PCS was 49.8 ± 11.9 in YG and 48.2 ± 9.5 in EG ( *P = 0.61)*. The postoperative MCS was also comparable between the two groups (48.4 ± 10.7 in YG vs. 50.1 ± 6.9 in EG; *P = 0.54*).

## Discussion

Surgical treatment in the so called "elderly" patients has been extensively reported even for oncological diseases either by laparotomy or laparoscopy [28,29].

Several studies showed laparoscopic surgery to be a safe and effective treatment for GERD being able to improve quality of life and warranting an early return to daily activities [30,31]

In elderly population with GERD, laparoscopic surgery has proven to be effective with low morbidity and mortality rates [32].

Previous study have shown comparable similar surgical results between the elderly group and nonelderly group including similar operation time, postoperative hospital stay, and low morbidity and mortality[33,34]. Our study also demonstrated similar results in terms of postoperative complication rate. The finding that younger patients had shorter hospitalization and operative times is in contrast with a recent report that compared elderly to younger patients undergoing laparoscopic antireflux surgery [35].

Though we did not look specifically at costs, longer operative times and length of hospital stay presumably might have affected operative and hospital expenses. In the current climate of medical cost control and limited resources, even the importance of cost-effectiveness cannot be ignored when evaluating a surgical technique or procedure [36,37].

A significant postoperative improvement in heartburn, regurgitation, chest pain and respiratory complications of GERD was observed in both EG and YG.

An excellent outcome after treatment was observed in a very high percentage of patients in both groups. A poor outcome was observed in those with a persistent postoperative dysphagia

A previous study showed laparoscopic total fundoplication is a safe and effective surgical treatment for gastroesophageal reflux disease generally offering an improved long-term quality of life, with the exception of a minority of patients who experience persistent severe dysphagia [38]. Authors suggested these patients may be offered the opportunity to undergo a laparoscopic redo fundoplication to treat the residual symptom, with encouraging results.

The current study employed the SF-36 as a well-validated and reliable health status measure widely used in medical and health services assessing the generic (ie, not diseasespecific) quality of life. In particular, patients in both groups improved in 3 scale scores that represent physical health: these measured physical function, role-limitation physical, and bodily pain and also in a scale score that normally is classified as a measure of mental health: vitality (energy and fatigue). In addition, the other 4 scale scores improved after laparoscopic total fundoplication without reaching statistical significance. There were also no significant difference in postoperative PCS and MCS scores based on the SF-36 between younger and elderly at long term follow up.

At last, we found overall outcome for patients who underwent surgery primarily for reflux was very good, with a very high satisfaction rate resulting comparable between younger and elderly

This finding is in keeping with previous studies stressing age should not be considered a contraindication to laparoscopic antireflux surgery that is a good option for the treatment of severe GERD also in octo- and nonagenarians [39]. The present study is potentially limited by the exclusive use of a general measure of health status. The additional use of a disease specific quality of life instrument has been recommended by the European Study Group for Antireflux Surgery [40,41]. This is important, especially if research's object is to adequately focus on an area of interest such as severity of symptoms as well as on response to treatment [42-44]. At last, this is a single tertiary referral center study and does not allow determination of the long-term outcome in a community practice setting.

In conclusion, laparoscopic total fundoplication is a safe and effective surgical treatment for gastroesophageal reflux disease generally warranting low morbidity and mortality rates and a significant improvement of symptoms comparable. An improved long-term quality of life is warranted even in the elderly.

## List of abbreviations used

Gastro-Esophageal Reflux Disease (GERD); Elderly Group (EG); Young Group (YG); Quality of life (QOL); Physical Component Summary (PCS); Mental Component Summary (MCS)

## Competing interests

The authors declare that they have no competing interests.

## Authors' contributions

ST, GDG, GD have made substantial contributions to conception and design; LB, IV, SG have made substantial contributions to acquisition of data; CV, ADA, GC have made substantial contributions to analysis and interpretation of data; FSL, NL, RP have been involved in drafting the manuscript; LD, GD, RR have been involved in revising it critically for important intellectual content; ST, GD, RR have given final approval of the version to be published.

## Authors' information

ST Research Grant Fellow at Second University of Naples; GD Associate Professor of Surgery at Second University of Naples; GDG Assistant Professor of Surgery at Second University of Naples; LB Senior Doctor at Second University of Naples; IV Senior Doctor at Second University of Naples; SG Research Fellow at Second University of Naples; CV Medical Student at Second University of Naples; ADA Surgical Fellow Resident at Second University of Naples; GC Surgical Fellow Resident at Second University of Naples; FSL Medical Doctor at Second University of Naples; NL Medical Student at Second University of Naples; RP Medical Student at Second University of Naples; RR Assistant Professor of Surgery at Second University of Naples; LD Full Professor of Surgery at Second University of Naples.

## Declarations

Funding for this article has come from personal funds.

This article has been published as part of *BMC Surgery *Volume 13 Supplement 2, 2013: Proceedings from the 26th National Congress of the Italian Society of Geriatric Surgery. The full contents of the supplement are available online at http://www.biomedcentral.com/bmcsurg/supplements/13/S2
